# Independent and joint effects of body mass index and metabolic health in mid- and late-life on all-cause mortality: a cohort study from the Swedish Twin Registry with a mean follow-up of 13 Years

**DOI:** 10.1186/s12889-022-13082-3

**Published:** 2022-04-11

**Authors:** Peggy Ler, Xia Li, Linda B. Hassing, Chandra A. Reynolds, Deborah Finkel, Ida K. Karlsson, Anna K. Dahl Aslan

**Affiliations:** 1 Aging Research Network-Jönköping (ARN-J), School of Health and Welfare, Jönköping University, Jönköping, Sweden; 2 Department of Medical Epidemiology and Biostatistics, Karolinska Institutet, Stockholm, Sweden; 3 Department of Psychology and Centre for Ageing and Health, University of Gothenburg, Gothenburg, Sweden; 4 Department of Psychology, University of California - Riverside, Riverside, CA USA; 5 Department of Psychology, Indiana University Southeast, New Albany, Indiana USA; 6 School of Health Sciences, University of Skövde, Skövde, Sweden

**Keywords:** Metabolic syndrome, Body weight, Obesity, Metabolically healthy obesity, Metabolically benign obesity, Mortality

## Abstract

**Background:**

There is robust evidence that in midlife, higher body mass index (BMI) and metabolic syndrome (MetS), which often co-exist, are associated with increased mortality risk. However, late-life findings are inconclusive, and few studies have examined how metabolic health status (MHS) affects the BMI–mortality association in different age categories. We, therefore, aimed to investigate how mid- and late-life BMI and MHS interact to affect the risk of mortality.

**Methods:**

This cohort study included 12,467 participants from the Swedish Twin Registry, with height, weight, and MHS measures from 1958—2008 and mortality data linked through 2020. We applied Cox proportional hazard regression with age as a timescale to examine how BMI categories (normal weight, overweight, obesity) and MHS (identification of MetS determined by presence/absence of hypertension, hyperglycemia, low HDL, hypertriglyceridemia), independently and in interaction, are associated with the risk of all-cause mortality. Models were adjusted for sex, education, smoking, and cardiovascular disease.

**Results:**

The midlife group included 6,252 participants with a mean age of 59.6 years (range = 44.9—65.0) and 44.1% women. The late-life group included 6,215 participants with mean age 73.1 years (65.1—95.3) and 46.6% women. In independent effect models, metabolically unhealthy status in midlife increased mortality risks by 31% [hazard ratio 1.31; 95% confidence interval 1.12–1.53] and in late-life, by 18% (1.18;1.10–1.26) relative to metabolically healthy individuals. Midlife obesity increased the mortality risks by 30% (1.30;1.06–1.60) and late-life obesity by 15% (1.15; 1.04–1.27) relative to normal weight. In joint models, the BMI estimates were attenuated while those of MHS were less affected. Models including BMI-MHS categories revealed that, compared to metabolically healthy normal weight, the metabolically unhealthy obesity group had increased mortality risks by 53% (1.53;1.19—1.96) in midlife, and across all BMI categories in late-life (normal weight 1.12; 1.01–1.25, overweight 1.10;1.01–1.21, obesity 1.31;1.15–1.49). Mortality risk was decreased by 9% (0.91; 0.83–0.99) among those with metabolically healthy overweight in late-life.

**Conclusions:**

MHS strongly influenced the BMI-mortality association, such that individuals who were metabolically healthy with overweight or obesity in mid- or late-life did not carry excess risks of mortality. Being metabolically unhealthy had a higher risk of mortality independent of their BMI.

**Supplementary Information:**

The online version contains supplementary material available at 10.1186/s12889-022-13082-3.

## Background

From 1980 to 2015, the prevalence of obesity, commonly defined as a body mass index (BMI) of 30 kg/m^2^ or greater, doubled among adults in more than 70 countries [1]. Excessive BMI is a putative risk factor for several major non-communicable diseases that are the principal causes of mortality. In 2015, an estimated four million deaths worldwide have been attributed to overweight, defined as BMI exceeding 25 kg/m^2^, 61% of which were related to obesity [1]. Hence, the growing obesity epidemic raises critical public health concerns.

Several multi-country, population-based studies, with research participants of a wide age range between 20 and 80 years, have shown that high BMI increases mortality risk [2–5]. However, in age-stratified analyses, the BMI-mortality association among older persons weakened [3–6], in agreement with other studies of older individuals, which have reported a nonsignificant or even inverse association between high BMI in late-life and mortality [7–10]. Therefore, the effects of high BMI in late-life on mortality remain unclear.

High BMI often clusters together with other cardio-metabolic factors, namely hypertension, dyslipidemia, and hyperglycemia, in what is known as metabolic syndrome (MetS) [11]. The prevalence of MetS is rising, resembling the obesity epidemic [12]. Although extensive research has demonstrated MetS as a risk factor for mortality, the evidence is primarily substantiated by studies of individuals less than 65 years [13, 14]. Some studies of older individuals, in contrast, have demonstrated a nonsignificant association between MetS and all-cause mortality [15, 16]. These findings suggest that the MetS-mortality association, like the BMI-mortality association, is likely moderated by age.

While high BMI often presents with MetS, they do not always co-exist. Obesity with the absence of MetS, signifying the preservation of metabolic health status (MHS), is referred to as metabolically healthy obesity (MHO) [17–20]. Interestingly, the MHO phenotype is not uncommon, and the prevalence of MHO in a population with obesity from 10 cohorts in Europe ranged from 35 to 76% in females and 22% to 57% in males [21]. Whether the MHO phenotype is a benign condition that confers lower mortality risk is, however, debatable. Some studies have demonstrated that MHO is associated with an elevated risk of mortality [22–24], but others have not [23, 25]. Furthermore, the mean ages of the study populations are generally less than 55 years, with follow-up ranging from about three to 30 years [24]. Research investigating the association between BMI-MHS phenotypes and mortality is scarce, especially in old age.

The increasing number of older individuals and the escalating prevalence of high BMI and MetS underscores the importance of elucidating how high BMI, MHS and their interactions predisposes individuals to mortality. Therefore, we aimed to examine the independent and joint effects of BMI and MHS on the risk of all-cause mortality and investigate age-specific effects by studying the associations separately in measures taken in midlife and late-life.

## Methods

### Study population

**Fig. 1:**
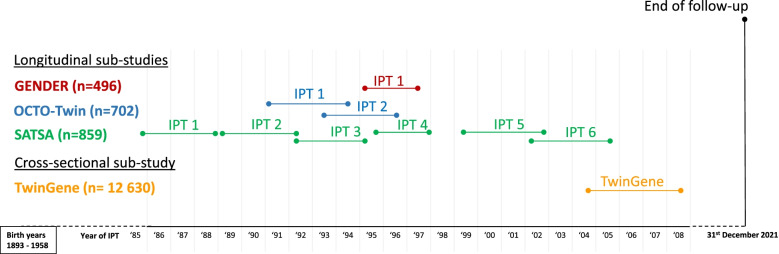
Timeline displaying when baseline measures were collected in study population from four sub-studies within the Swedish Twin Registry. The birth year of the participants included are depicted within the box on the left of the figure. The timeline above depicts the year when IPTs occurred and when baseline measures were derived. The four sub-studies are listed in the left column. n denotes the total number of participants for each sub-study before implementing exclusions in this current study. IPT indicates the in-person testing phase relevant for the present study

The is a prospective, cohort study including participants from four sub-studies of aging in the Swedish Twin Registry, born from 1893 to 1958 [26]: GENDER [27], OCTO-Twin [28], SATSA [29] and TwinGene [30]. GENDER, OCTO-Twin and SATSA are longitudinal studies where various health status measures were collected in phases of self-reported questionnaires and in-person testing (IPT). The IPT included assessments of weight, height, blood pressure (BP) and venous blood sample collection. Licensed nurses performed the IPT at the participants’ homes or local healthcare centres. TwinGene is a cross-sectional study where participants underwent a health examination and venous blood sample collection at a local health care facility between 2004 and 2008. In this present study, the baseline was the first IPT with venous blood sample collection performed. Therefore, the baseline of this current study was between 1985 and 2008 (Fig. 1), which may occur at IPTs for different participants, depending on their age and wave of entry into the study, i.e., the study period spanned from baseline ranging from 1985 to 2008 to death or December 31st, 2020, whichever came first. All participants provided written informed consent, and the study was approved by Ethical Review Board in Stockholm (2015/1729 – 35/5).

### Assessing body mass index and metabolic health status

Height and weight were measured in light clothes (where participants were asked to remove their shoes, heavy items, or clothing) by trained nurses during the IPT. Systolic and diastolic BP was measured twice after 5 min of rest. Serum levels of glucose, haemoglobin A1C (HbA1c), triglycerides (TG) and high-density lipoproteins cholesterol (HDL-C) were analyzed from fasting (92.1%) and non-fasting (7.9%) blood samples. Diagnoses of diabetes, the use of diabetic medications and lipid-lowering medications were obtained from self-reported questionnaires or interviews during the IPT.

BMI was calculated by dividing weight in kilograms (kg) by the square of height in meters (m^2^) and categorized according to the WHO criteria [31] as follows: underweight (< 18.5), normal weight (18.5 – 24.9 kg/m^2^), overweight (25.0 – 29.9 kg/m^2^) and obese (≥ 30.0 kg/m^2^). Since few participants were underweight (*n* = 170, 1.3%), they were excluded from the analyses. Weight history was the maximum BMI recorded between ages 45 and 65, at a minimum of 5 years before the study baseline. The source of weight history may be IPT or self-reported from questionnaires. Weight history was categorized into normal weight, overweight or obese according to the WHO criteria mentioned above.

The National Cholesterol Education Program Adult Treatment Panel-III (NCEP ATP-III) criteria for MetS [32], previous literature [17, 21], and data availability guided the ascertainment of MHS in this study. Metabolically unhealthy (MU) status was defined as having at least two of the following NCEP ATP-III metabolic components: hypertension, hyperglycemia, hypertriglyceridemia, and decreased HDL. Table 1 shows the details of thresholds for each metabolic parameter. The presence of only one or none of the above indicated being metabolically healthy (MH).

**Table 1:** Criteria and thresholds for defining metabolically unhealthy status

Metabolic components	Criteria and Thresholds
Hypertension	• Systolic BP ≥ 135 mmHg, or • Diastolic BP ≥ 85 mmHg
Hyperglycaemia	• Fasting BG ≥ 6.1 mmol/L, or • Non-fasting BG ≥ 7.0 mmol/L, or • HbA1c ≥ 5.7%, or • Self-reported use of diabetic medications, or • Self-reported diagnosis of diabetes
Hypertriglyceridemia	• Fasting TG ≥ 1.70 mmol/L, or • Non-fasting TG ≥ 2.1 mmol/L, or • Self-reported use of lipid-lowering medications
Decreased HDL-C	• < 1.03 mmol/L in males, and • < 1.30 mmol/L in females, or • Self-reported use of lipid-lowering medications

The presence of ≥ 2 metabolic components determines metabolically unhealthy status. The presence of a metabolic component is defined by meeting any one threshold or criteria. Abbreviations—*BP* blood pressure, *BG* blood glucose, *HbA1c* haemoglobin A1C, *TG* triglyceride level, *HDL-C* high-density lipoproteins cholesterol

Studying the interaction between BMI and MHS generated six different BMI-MHS phenotypes: metabolically healthy normal weight (MHN), metabolically unhealthy normal weight (MUN), metabolically healthy overweight (MHOw), metabolically unhealthy overweight (MUOw), metabolically healthy obesity (MHO) and metabolically unhealthy obesity (MUO).

### Covariates

Education (≤ 7 years, > 7 years, corresponding to basic versus more than a basic education for these birth cohorts) and smoking status (ever-smoker, never smoker) were self-reported during the studies. History of cardiovascular disease (CVD) was based on self-reports of angina pectoris, myocardial infarction, hypertension, angina, thrombosis of the legs, ischemic stroke or hemorrhagic stroke.

### All-cause mortality

The STR is linked to several nationwide registers, including the Swedish Tax Agency, where information about participants’ vital status (the state of being alive or dead) and date of death was obtained. The outcome of interest was all-cause mortality, i.e., death due to any reason. Participants were followed from the age when BMI and MHS were assessed until death or December 31^st^, 2020, depending on which came first.

### Statistical analyses

We applied Cox proportional hazards regression to estimate hazard ratios (HR) and 95% confidence intervals (CI) of the individual and joint effects of BMI and MHS on the risk of all-cause mortality, with age as the underlying time scale. We selected age as a timescale in our analyses since age strongly correlates to the exposure of interest (BMI and MHS) and survival. Using age as a timescale allows us to account for the effects of age at study entry when exposures were measured, age at the end of the study, and age at death.

Participants were followed from the age of BMI and MHS assessment to death or end of follow-up (December 31^st^, 2020). We used stratified Cox models to account for differences among the sub-studies and robust standard errors to account for clustering within twin pairs. We divided the study population into two groups based on age at baseline in the analyses. The midlife group consisted of participants whose BMI and MHS were measured at ages 65 years or below (≤ 65.0 years), while the late-life group consisted of participants whose BMI and MHS were measured at ages greater than 65 years (> 65.01 years). The proportional hazards assumption was tested by comparing log–log survival plots and performing tests on Schoenfeld residuals for each independent variable in the main analysis. CVD in midlife and smoking in late-life variables did not satisfy the proportional hazard assumption and were thus specified as time-varying covariates.

Firstly, we examined the independent effects of BMI by estimating the association adjusted for the primary confounders, education, smoking and sex (Model 1), and further for CVD (Model 2). Then, the independent effects of MHS on the risk of mortality were investigated with the corresponding two models mentioned above (Model 3 and 4). Next, we estimated the joint effects of both BMI and MHS, adjusted for the primary confounders as above (Model 5), and further adjusted for CVD (Model 6).

Lastly, we included an interaction term between BMI and MHS, adjusted for primary confounders (Model 7) and further for CVD (Model 8), to study how BMI stratified by MHS was associated with mortality. We thereby estimated HRs with 95% CI for mortality of the six phenotypes generated from the cross-categorization of BMI and MHS: MUN, MHOw, MUOw, MHO and MUO with MHN as the reference category. The normative groups (MHN, normal weight, and metabolically healthy category) were selected as the reference category in line with previous research.

Sensitivity analyses were performed based on models 7 and 8, using the full sample, first by adjusting for education, smoking and sex, then further adjusted for CVD. Since there is no consensus in the criteria for ascertaining metabolic health, we first investigated if the HRs and 95% CI were affected by applying five different ways of determining MHS: a) by including CVD history as an additional criterion in the definition; b) excluding self-reports, such as the use of diabetic medications, lipid-lowering medications and diabetes diagnosis; c) defining MH status as the absence of any metabolic abnormality; d) including waist circumference(WC) as an additional criterion in the definition. The thresholds for WC to indicate metabolic abnormality were 80 cm for females and 94 cm for males; e)we applied the new criteria established by Zembic et al. [33] and defined MH as the absence of the following: 1) systolic BP less than 130 mmHg, 2)waist-hip-ratio (WHR) less than 0.95 for women and less than 1.03 in men, 3) no prevalent diabetes. Secondly, we added weight history as a confounder to correct for potential bias from reverse causation. Next, we stratified the analyses by sex to detect sex differences in the BMI-MHS-mortality association. Lastly, we examined the individual effects of metabolic abnormalities used to define MHS by including them separately within the same model. All analyses were performed with STATA version 16.1.

## Results

### Baseline characteristics

After excluding participants with insufficient information to define any one of the four metabolic components (*n* = 598, 4.5%) or missing covariates (*n* = 87, 0.6%), we included 12,467 individuals in the analyses with a mean follow-up of 13 years (range 0.01 – 33.7). The mean follow-up time was derived from a study period that spanned from baseline ranging from 1985 to 2008 to death or December 31st, 2020, whichever came first. Table 2 summarizes the baseline characteristics of the study population. A total of 6,252 participants entered the study in midlife, and 6,215 participants entered in late-life. The mean baseline age and follow-up time were 59.6 (range 44.9 – 65.0) and 13.9 (1.0 – 33.7) years respectively in the midlife group and 73.1 (65.1 – 95.3) and 12.0 (0.01–30.8) years in the late-life group. The mean BMI was approximately 26 kg/m^2^ at baseline in both the midlife and late-life sample, while the prevalence of MU status was higher in late-life. During follow-up, 733 deaths occurred in the midlife group at a mean age of 72.6 (60 – 96) years, and 3419 in the late-life group at a mean age of 85.2 (67 – 108) years.

**Table 2:** Descriptive statistics of baseline characteristics of study participants by age at baseline, midlife and late-life

**Characteristics**	Age categories			
	**Midlife (< = 65 years)**		**Late-life (> 65 years)**	
	**Mean / n**	**SD / %**	**Mean / n**	**SD / %**
Number of participants (n)	6252		6215	
Age at baseline, years (mean, SD)	59.6	4.18	73.1	5.93
Sex, Males/Females (n, %)	2754/3498	44.05/55.95	2897/3318	46.61/53.39
BMI, kg/m2 (mean, SD)	26.06	3.94	26.01	3.81
Systolic BP, mmHg (mean, SD)	134.99	18.33	147.30	20.84
Diastolic BP, mmHg (mean, SD)	82.67	10.52	81.57	10.64
Blood glucose, mmol/l (mean, SD)				
Non fasting (*n* = 387)	4.88	1.33	5.30	2.13
Fasting (*n* = 400)	4.63	1.17	4.89	2.31
HbA1c, % (mean, SD)	4.75	0.65	4.90	0.69
Type II Diabetes (n, %)	300	4.82	555	8.98
Diabetic medication use (n, %)	223	3.57	401	6.46
Triglycerides, mmol/l (mean, SD)				
Non fasting (*n* = 506)	1.63	1.20	1.78	0.97
Fasting (*n* = 11,016)	1.34	0.84	1.40	0.79
HDL cholesterol, Males/Females, mmol/l (mean, SD)	1.23/1.57	0.34/0.42	1.25/1.55	0.34/0.42
Lipid-lowering medication use (n, %)	695	11.16	875	14.14
Education, < = 7 years/ > 7 years (n, %)	1553/4699	24.84/75.16	2742/3473	44.12/55.88
Ever smoker (n, %)	3943	63.07	3172	51.04
CVD (n, %)	2021	32.33	2900	46.66
BMI categories (n, %)				
Normal weight	2786	44.56	2677	43.07
Overweight	2581	41.28	2701	43.46
Obesity	885	14.16	837	13.47
Metabolically unhealthy (n, %)	2304	36.85	2831	45.55
No. of Metabolic abnormalities (mean, SD)	1.34	1.06	1.63	1.01
Mortality (n, %)	733	11.72	3419	55.01
BMI * Metabolic Health Status (%)				
MHN	2178	34.84	1800	28.96
MUN	608	9.72	877	14.11
MHOw	1454	23.26	1282	20.63
MUOw	1127	18.03	1419	22.83
MHO	316	5.05	302	4.86
MUO	569	9.10	535	8.61
Weight History (%)				
Normal weight	3259	56.19	2617	58.14
Overweight	2107	36.33	1616	35.90
Obesity	434	7.48	268	5.95

Baseline characteristics are presented as means and standard deviations (SD) for continuous variables; and frequencies (n) and percentages (%) for categorical variables. Individuals with measures taken in mid-and late-life are presented separately

Abbreviations: *BMI* body mass index, *BP* blood pressure, *CVD* history of cardiovascular disease or cardiovascular surgeries, *MHS* metabolic health status, *MHN* metabolically healthy normal weight, *MUN* metabolically unhealthy normal weight, *MHOw* metabolically healthy overweight, *MUOw* metabolically unhealthy overweight, *MHO* metabolically healthy obesity, *MUO* metabolically healthy obesity. Normal weight was defined as having BMI 18.5 – 24.9 kg/m^2^; overweight 25 – 29 kg/m^2^; obesity ≥ 30 kg/m^2^. Metabolically unhealthy status was defined as having ≥ 2 abnormal metabolic abnormalities. Metabolically healthy status was defined as having < 2 abnormal metabolic components

### Independent and joint effects of BMI and MHS on the risk of mortality

Table 3 shows how mid- and late-life BMI and MHS associate with mortality, independently and jointly, adjusted for the primary confounders, education, smoking, and sex, and further adjusted for CVD. Mid- and late-life obesity, but not overweight, were associated with 42% and 22% higher risk of mortality, respectively, compared to normal weight. Further adjustments for CVD attenuated the effects of midlife obesity to 30% and late-life obesity to 15%. Compared to the MH group, being MU in midlife was associated with a 43% rise in mortality risk and being MU in late-life was associated with 25% higher risk. Further adjustments for CVD slightly attenuated the effects of the MU group in mid- and late-life to 31% and 18%, respectively.

**Table 3:** Multivariable Cox regression of all-cause mortality in relation to independent and joint effects of body mass index and metabolic health status

Age	Models	Independent effects of BMI		Independent effects of MHS		Joint effects of BMI and MHS	
		Model 1	Model 2	Model 3	Model 4	Model 5	Model 6
		HR (95% CI)	HR (95% CI)	HR (95% CI)	HR (95% CI)	HR (95% CI)	HR (95% CI)
	**Adjustments**	Sex, Educ, Smoke	+ CVD	Sex, Educ, Smoke	+ CVD	Sex, Educ, Smoke	+ CVD
** < = 65 years**	**BMI categories**						
	**Overweight**	1.02 (0.87 – 1.21)	0.99 (0.84 – 1.17)			0.96 (0.81 – 1.13)	0.94 (0.80 – 1.12)
	**Obesity**	**1.42 (1.16 -1.75)**	**1.30 (1.06 – 1.60)**			**1.25 (1.003 – 1.55)**	1.19 (0.96 – 1.48)
	**MU status**			**1.43 (1.23 – 1.66)**	**1.31 (1.12 – 1.53)**	**1.38 (1.18 – 1.62)**	**1.28 (1.09 – 1.51)**
** > 65 years**	**BMI categories**						
	**Overweight**	0.99 (0.93 – 1.07)	0.96 (0.89 – 1.03)			0.96 (0.89 – 1.03)	0.94 (0.87 – 1.01)
	**Obesity**	**1.22 (1.10 – 1.35)**	**1.15 (1.04 – 1.27)**			**1.15 (1.04 – 1.28)**	**1.11 (1.01 – 1.23)**
	**MU status**			**1.25 (1.17 – 1.34)**	**1.18 (1.10 – 1.26)**	**1.25 (1.16 – 1.33)**	**1.18 (1.10 – 1.26)**

Hazard ratios with 95% confidence intervals from Cox regression models of all-cause mortality in relation to independent effects of body mass index (BMI), metabolic health status (MHS) and joint effects of BMI and MHS. Models 1, 3 and 5 were adjusted for sex, education attainment and smoking status. Models 2, 4 and 6 were adjusted for sex, education attainment, smoking status and history of cardiovascular disease. Bold numbers indicate significance at the α = 0.05 level

Abbreviations: *HR* hazard ratios, *CI* confidence interval, *Educ* education attainment, *Smoke* smoking status, + *CVD* additionally adjusted with history of cardiovascular disease, *BMI* body mass index, *MU* metabolically unhealthy

In the joint models, which include the effects of both BMI and MHS, we observed the most substantial differences in the HRs for obesity, which was attenuated to 25% when measured in midlife and 15% when measured in late-life. When further adjusted for CVD, the pattern of change in the independent effects and joint effects were identical. Notably, the association between midlife obesity and mortality became nonsignificant when CVD was added to the models. The HRs for MU in both midlife and late-life were generally similar in the independent effect and joint models. In the joint effect models, being MU in midlife was associated with an elevated mortality risk by 38% and in late-life by 25%. When further adjusted for CVD, midlife and late-life MU was associated with increased mortality risk by 28% and 18%, respectively.

### Interaction between BMI and MHS in relation to the risk of mortality

**Fig. 2:**
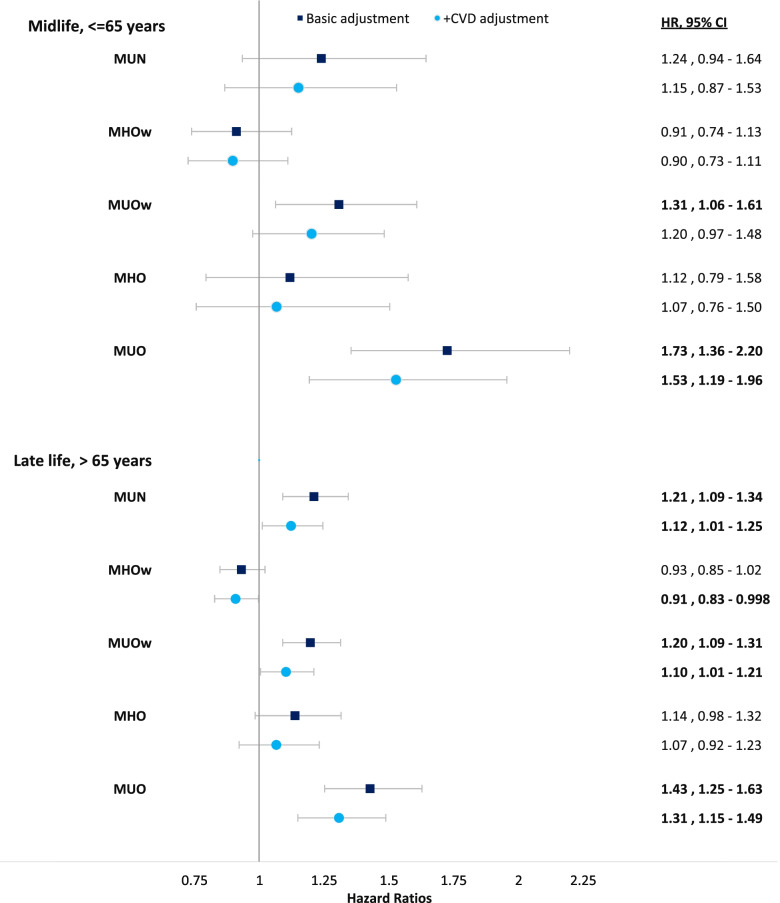
Multivariable Cox Regression of all-cause mortality in relation to the interactions between body mass index and metabolic health status. Hazard ratios (HR) and 95% confidence intervals (CI) of all-cause mortality in relation to the interactions between body mass index (BMI) categories and metabolic health status, with metabolically healthy normal weight (MHN) as the reference group. We present Cox regression models adjusted for sex, education, and smoking; and with + CVD adjustment adjusted for sex, education, smoking and CVD. Bold numbers denote significance at the α ≤ 0.05 level. Abbreviations: CVD—history of cardiovascular disease, MUN – metabolically unhealthy normal, MHOw – metabolically healthy overweight, MUOw – metabolically unhealthy overweight, MHO – metabolically healthy obese and MUO – metabolically unhealthy obese. Normal weight is defined as having BMI 18.5 – 24.9 kg/m^2^; overweight 25 – 29.9 kg/m^2^; obesity ≥ 30.0 kg/m^2^. MU is defined as having ≥ 2 abnormal metabolic abnormalities

Figure 2 presents the HRs and 95% CIs of BMI-MHS phenotypes, generated from the interaction between BMI and MHS, with MHN as the reference group for all-cause mortality. When adjusted for primary confounders, being MUOw or MUO in midlife heightened the risk of mortality by 31% and 73%, respectively. In contrast, being MHOw or MHO was not associated with a rise in mortality risk. After additional adjustment of CVD, the HR for MUOw in midlife was attenuated and no longer significant; however, MUO was associated with a higher mortality risk at 53%.

In late-life, being MU increased mortality risk across all BMI categories compared to MHN (Fig. 2). Relative to MHN, the risk of mortality increased by 21% in MUN, 20% in MUOw, and 43% in MUO. When including CVD in the adjustment, HRs were slightly attenuated for all the MU phenotypes. Being MHOw in late-life was associated with a 9% lower risk of mortality. While individuals with MHO in late-life had higher HRs for mortality in all models, they were not statistically significant.

### Sensitivity analyses

There were no striking sex differences in the findings, but the HRs were generally higher among males than females, particularly in midlife (Additional file 1, Tables S7a and S7b).

Overall, using other definitions of MHS affected the magnitude of effects but not the pattern or general conclusions (Additional file 1, Table S1 – S6). When we added CVD as an additional criterion in the assessment of MHS, the association between BMI-MHS phenotypes and mortality became stronger (Additional file 1, Table S1). Contrarily, when self-reports such as medication use and diabetes diagnosis were excluded from the assessment of MHS, the magnitude of the associations were weakened, and the negative association between MHOw and mortality in midlife became nonsignificant (Additional file 1, Table S2). If MH status was defined as the absence of any cardiometabolic abnormalities, MHO approximately doubled the risk for mortality in mid- and late-life compared to MHN (Additional file 1, Table S3). However, the low numbers of individuals with MHO may have contributed to the exceptionally high HRs for being MHO. Adding WC in the assessment of MHS did not affect the results qualitatively (Additional file 1, Tables S4 and S5).

**Fig. 3:**
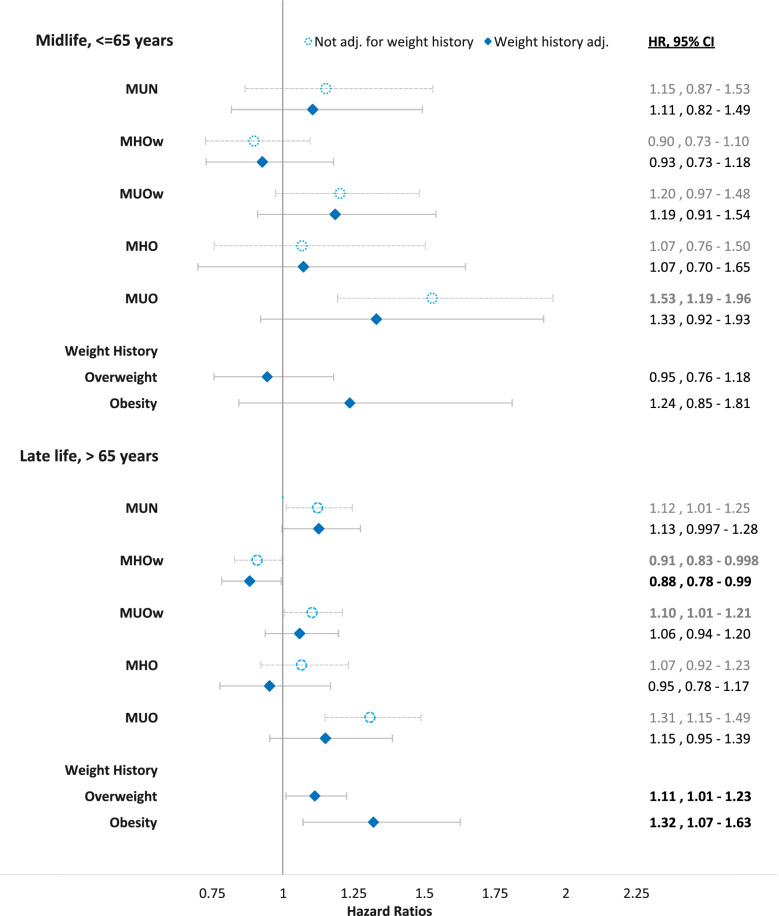
Multivariable Cox Regression of all-cause mortality in relation to the interactions between body mass index and metabolic health status, not adjusted for weight history versus adjusted for weight history. Hazard ratios (HR) and 95% confidence intervals (CI) of all-cause mortality in relation to interactions between body mass index categories (BMI) and metabolic health status adjusted for weight history. We present Cox regression models adjusted for education, smoking, sex, and CVD, without and with weight history. Reference group is MHN – metabolically healthy normal weight. Bold numbers denote significance at the α = 0.05 level. Abbreviations: CVD—history of cardiovascular disease, MUN – metabolically unhealthy normal weight, MHOw – metabolically healthy overweight, MUOw – metabolically unhealthy overweight, MHO – metabolically healthy obesity and MUO – metabolically unhealthy obesity. Normal weight is defined as having BMI 18.5 – 24.9 kg/m^2^; overweight 25 – 29.9 kg/m^2^; obesity ≥ 30 kg/m^2^. MU is defined as having ≥ 2 abnormal metabolic abnormalities

Figure 3 compares the changes in estimates of BMI-MHS interactions for all-cause mortality from Model 8 (adjusted for primary covariates and CVD) with the model further adjusted for weight history. There were no statistically significant findings in the midlife sample. Neither a weight history of overweight nor obesity in midlife was associated with higher mortality. The elevated risk of mortality related to MUO in midlife became insignificant when further adjusted with weight history. In the late-life sample, a weight history of being overweight and obese was associated with an 11% and 32% increase in mortality risk. Further adjustment with weight history rendered the positive association of late-life MUOw and MUO with mortality risk insignificant. However, the higher HRs associated with late-life MUN and decreased HR associated with late-life MHOw remained robust to the adjustment.

Table 4 presents how the individual metabolic parameters used to define MHS relate to all-cause mortality. Out of the four metabolic parameters used to determine MHS, hyperglycemia had the largest effect size, increasing the mortality risk by 78% in the midlife group and 52% in the late-life group compared to normoglycemia. Hypertriglyceridemia in late-life was associated with a 9% increase in mortality, but the HR was not significant in midlife. Hypertension, BMI, and low HDL-C were not significantly associated with all-cause mortality, regardless of age at measurement. CVD in midlife was strongly associated with mortality risk, increasing the risk of mortality by nine times compared to those without a history of CVD, but with a time-varying effect such that HRs decreased by 3% per year of survival. In late-life, CVD was associated with a 33% increase in mortality risk.

**Table 4:** Multivariable Cox Regression of all-cause mortality in relation to individual metabolic abnormalities used to define metabolic health status

Age	Metabolic Abnormalities	HR	95% CI
** < = 65 years** (*n* = 6252)	BMI categories		
	Normal weight	Ref	
	Overweight	0.95	0.80—1.13
	Obesity	1.13	0.90—1.42
	Hypertension	1.00	0.85—1.19
	Hyperglycaemia	**1.78**	**1.43—2.20**
	Hypertriglyceridemia	1.13	0.94—1.36
	Low HDL-C	1.01	0.83—1.22
	CVD History	**8.94**	**2.06 – 38.81**
	CVD*t	**0.97**	**0.95 – 0.99**
** > 65 years** (*n* = 6215)	BMI categories		
	Normal weight	Ref	
	Overweight	0.93	0.87—1.00
	Obesity	1.10	0.99—1.21
	Hypertension	0.93	0.85—1.04
	Hyperglycaemia	**1.52**	**1.38—1.67**
	Hypertriglyceridemia	**1.09**	**1.01—1.18**
	Low HDL-C	1.02	0.94—1.11
	CVD History	**1.33**	**1.25—1.43**

Hazard ratios with 95% confidence intervals of all-cause mortality in relation to BMI category and individual metabolic abnormalities, adjusted for sex, education, and smoking

Abbreviations: *HR* hazard ratio, *CI* confidence interval, *n* sample size, *Ref* reference group, *BMI* body mass index, *HDL-C* high-density lipoprotein cholesterol levels, *CVD* cardiovascular disease, CVD*t CVD as a time-varying covariate

## Discussion

### Summary of findings

To the best of our knowledge, this is the first study that examined both the independent and joint effects of BMI and MHS and their interactions on mortality in the same study population stratified into mid- and late-life. In this cohort of 6,252 individuals with measures taken in midlife, and 6,215 individuals with measures taken in late-life, being MU, is independently associated with an elevated risk of mortality, irrespective of BMI. Midlife and late-life overweight and obesity is associated with increased mortality risk only among those who were MU. Conversely, metabolically healthy overweight in late-life is associated with a reduced risk of mortality.

### Independent effects of BMI and MHS in mid- and late-life

From the independent effect models, obesity, but not overweight, in both mid- and late-life was associated with increased mortality risk. The midlife results are consistent with other large cohort studies [4, 5], but the positive association between late-life obesity and mortality contrasts with many studies on older persons [2, 6–9]. Being MU in either age group was also associated with an elevated risk of mortality, aligning with meta-analyses on mortality risks associated with MetS in a population with a broad spectrum of ages [13] and older persons [34]. While the direction of the independent effects of high BMI and MU status on mortality was broadly similar in mid- and late-life, the magnitude of the effects from obesity and being MU in midlife were greater than in late-life. These findings highlight the importance of initiating prevention and interventions to manage obesity and metabolic dysfunction early in adulthood due to its potential long-term impact on survival.

### Comparing the independent and joint effects of BMI and MHS

The effects of MHS on mortality remained relatively stable in the joint effect models, whereas the effects of obesity on mortality were attenuated compared to the independent effect models. Therefore, MHS is a stronger predictor of mortality than obesity in both mid- and late-life. In the interaction models, MU individuals had higher risks of mortality than the MH group, regardless of BMI category. Furthermore, individuals belonging to a lower BMI category who were MU carried higher mortality risks than those with a higher BMI and MH, consistent with past studies [35, 36]. A study conducted among older persons has also demonstrated that MetS accounted for 71.3% of a BMI and CVD association [37]. Collectively, these results suggest that MU status may be a primary driver of elevated mortality risk.

### Effects of BMI and MHS interactions

Even among individuals with normal weight in late-life, being MU (MUN) increased the risk of mortality compared to MHN, consistent with past research [35, 36, 38, 39]. The raised mortality risk observed among those with MUN in late-life may result from reverse causality—weight loss from pre-existing illnesses. However, our findings show that the increased mortality risk in MUN was only slightly attenuated upon adjusting for weight history. This indicates that weight loss from overweight or obesity may not be key drivers to the excess mortality risk in MUN in late-life. Since the metabolic dysfunction in MUN is hidden in plain sight, detecting this phenotype is likely challenging. Greater attention to the evaluation of MHS may be necessary to better assess mortality risks, even in older adults with normal weight.

While individuals with higher BMI were likely to present with unfavourable metabolic profiles, we still found that within the group of people with obesity, 35.7% of the midlife sample and 36.1% of the late-life sample were metabolically healthy. The existence and prognosis of MHO is a subject that is debated in the literature. Our findings strengthen the evidence of past research demonstrating MHO as a nonsignificant risk of mortality [36, 39, 40]. Nonetheless, other studies have reported an increased [38, 41] or decreased [35, 42] risk of mortality among individuals with MHO. Some have attributed the heterogeneous findings to the various criteria and thresholds used in determining MHS and have called for consensus in the definitions [17, 43].

### Criteria for defining MHS—findings from sensitivity analyses

It is important to note that only the magnitude of effects changed while the conclusions generally remained the same when various definitions of metabolic health were explored. One exception was the recently proposed criteria of MHS established systematically to distinguish MHO with decreased mortality by Zembic et al. [33]. Applying these new definitions did not substantially change our results in the midlife group (Additional file 1, Table S6). However, late-life MHO was significantly associated with a greater risk of mortality, corresponding with the findings in our sensitivity analysis, when metabolic health was defined with stricter criteria (absence of metabolic abnormality). It is noteworthy that the number of persons with MHO in our study dropped substantially when using the new definitions or stricter criteria, which may have explained the rise in mortality risks associated with MHO in late-life. Since these new criteria were derived systematically from a younger population with a mean age of 41.6 years, the risk pattern yielded from our midlife group was consistent with the study [33], as expected. Nonetheless, the findings from the late-life group were contradictory, thus raising concerns about whether criteria established in a midlife sample apply as well in late-life. Therefore, it may be justified to create age-specific criteria and cut-offs in the definitions of MHS and obesity.

### Effects of MHOw in particular

The obesity paradox, the counterintuitive lower risks of mortality among those with high BMI, which tends to manifest in studies among older persons [2, 6–9], was not observed in our study. However, the lack of association between those with MHO and mortality and the negative association between MHOw in late-life and mortality highlight the role of MHS in generating paradoxical relations between high BMI and mortality. For example, an obesity paradox may present in a study population where individuals with higher BMI were metabolically healthier. Such a selection bias for healthier individuals tend to occur in many studies on aging.

This decline in mortality risk associated with MHOw in late-life casts doubts on weight loss recommendations for older persons who are metabolically healthy and overweight. Indeed, the latest nutrition and hydration guidelines in geriatrics established by the European Society for Clinical Nutrition and Metabolism advocated against weight-reducing diets for older individuals who are overweight [44]. The authors mentioned the accumulating evidence of the importance of metabolic risks; notwithstanding, there were no recommendations in assessing MHS as part of risk evaluation related to BMI.

### The risk of mortality from individual metabolic components from sensitivity analysis

Among the metabolic components used to determine MHS, hyperglycaemia had the largest independent effects on mortality in both age categories, in line with previous literature [38, 45]. However, reports of how the rest of the metabolic components relate to mortality have been conflicting. While the association between hypertriglyceridemia in late-life and mortality in our study corresponds with the research findings derived from a younger study population [38], a meta-analysis found hypertriglyceridemia was protective among older persons with a median age of 73 years [45]. In our study, neither hypertension nor low HDL in mid- and late-life was associated with mortality, contradicting prior evidence [38, 45, 46]. The differences in how individual metabolic components relate to mortality may result from variations in age, pharmaceutical treatments, and the prevalence of metabolic dysfunctions in different study populations. Further investigations are necessary to understand this heterogeneity.

Although there is evidence that the effects of MHS are greater than BMI, the clinical importance of high BMI should not be downplayed. There is still substantial evidence linking high BMI to major non-communicable diseases, like CVD [47, 48], type II diabetes mellitus (T2DM) [48, 49] and cancer [50–52], which in turn predispose individuals to premature death. However, high BMI in itself may not be the proximal cause of mortality, thus explaining the weakened effects of BMI in the joint models and in interactions with MH status. Moreover, the dose-dependent increment in the prevalence of MU status among individuals from higher BMI categories in our study, in line with previous research [53], likely arises from the strong correlation between high BMI and metabolic dysfunction. Since metabolic parameters outside of the healthy range are well-recognized risk factors for CVD and T2DM [54], targeting both high BMI and impaired metabolic parameters is likely crucial in primary prevention.

### Strengths and limitations

These findings contribute to understanding the gaps in our knowledge of how mid- and late-life BMI and MHS, independently, jointly, and in interactions, impact mortality. In addition, weight and height used in the derivation of BMI were measured objectively by trained, licensed nurses, thus reducing measurement errors. Moreover, we included weight history in our models to limit reverse causality. Furthermore, since the outcomes data were obtained from linked Tax registries in Sweden, we had comprehensive coverage of mortality.

There are some limitations to our study. Firstly, we included thresholds on non-fasting glucose and lipid levels, which may underestimate the prevalence of hyperglycemia and dyslipidemia. However, the proportion of non-fasting measures was relatively low. Secondly, using age as a timescale in the analysis limits the ability to account for period effects, such as differences in medication use over time on the population level. Thirdly, the mean follow-up time of the midlife group, at 13.9 years, meant our data in the midlife group is at risk of capturing mostly early deaths. Since the sensitivity analyses indicated that CVD in midlife was associated with a nine-fold increase in the risk of death, premature death from CVD events likely accounted for most of the mortality in the midlife group. In addition, the unusually high HR of CVD may be an overestimation caused by the close link between CVD and MHS observed in other studies [55, 56]. Moreover, the estimates from the late-life group, with a mean follow-up time of 12 years, is potentially susceptible to reverse causation between lower BMI and mortality. Finally, to correct for potential bias due to weight loss from pre-existing morbidities, we adjusted the models for weight history, which did not drastically change the effects of BMI-MHS phenotypes.

Lastly, this study accounted for BMI and MHS only at baseline and could not capture the impact on mortality from the trajectories of BMI, MHS and the BMI-MHS phenotypes. When we included weight history in the models, the history of overweight and obesity heightened mortality risks, suggesting cumulative adverse effects from having high BMI. Furthermore, an extensive study of 90,257 women over 30 years supported the transient nature of BMI-MHS and showed that long periods of obesity increased CVD risk, despite preserved metabolic health [46]. The same study also concluded that many women with MHO transitioned to MUO over time. Future research should identify trajectories of BMI-MHS phenotypes and their impact on mortality.

## Conclusions

In this large, prospective study of BMI and MHS in relation to mortality, we demonstrated that a metabolically unhealthy status, both in mid- and late-life, is an independent risk factor for mortality, robust to adjustment for CVD as well as BMI category. On the contrary, the association between high BMI and mortality changes in dependence upon MHS. Specifically, MHS influences the BMI-mortality association, such that overweight or obesity is not associated with excess risks of mortality in individuals with preserved metabolic health. In fact, being MHOw in late-life was associated with lower mortality risks. Therefore, sole assessment of BMI is likely insufficient and more nuanced evaluations of BMI together with MHS can more critically assess an individual’s mortality risk.

## Supplementary Information

**Additional file 1:** **Additional files. Table S1.** Effects of bodymass index and metabolic health status interaction on all-cause mortality whenCVD was included as a component for ascertaining metabolic health. **Table S2.** Effects of bodymass index and metabolic health status interaction on all-cause mortality, whenself-reports were excluded from the ascertainment of metabolic health status. **Table S3.** Effects of bodymass index and metabolic health status interaction on all-cause mortality, whenmetabolic health is defined as the absence of any metabolic abnormality. **Table S4.** Effects of body massindex and metabolic health status interaction on all-cause mortality, when waistcircumference was included in the ascertainment of metabolic health status, andmetabolic health is defined as ≤ two metabolic abnormalities. **Table S5.** Effects of body massindex and metabolic health status interaction on all-cause mortality when waistcircumference is included in the ascertainment of metabolic health status, andmetabolic health is defined as ≤ three metabolic abnormalities. **Table S6.** Effects of body massindex and metabolic health status interaction on all-cause mortality, when MHS isdefined by new criteria [1], the absence ofhypertension, high waist-hip ratios and diabetes. **Table S7a. **Effects of mid-life bodymass index and metabolic health status interaction on all-cause mortality, stratifiedby sex. **Table S7b. **Effects of late-life bodymass index and metabolic health status interaction on all-cause mortality, stratifiedby sex. 

## Data Availability

The data that support the findings of this study are from the Swedish Twin Registry. Data can be applied for at https://ki.se/en/research/swedish-twin-registry-for-researchers.

## Abbreviations

BMI: Body mass index
BP: Blood pressure
CVD: History of cardiovascular disease
HDL-C: High-density lipoprotein cholesterol
HbA1c: Hemoglobin A1C
MetS: Metabolic Syndrome
MH: Metabolically healthy
MHS: Metabolic health status
MHN: Metabolically healthy normal weight
MHOw: Metabolically healthy overweight
MHO: Metabolically healthy obesity
MU: Metabolically unhealthy
MUN: Metabolically unhealthy normal weight
MUOw: Metabolically unhealthy overweight
MUO: Metabolically unhealthy obesity
TG: Triglyceride levels
