# Supplementary material for: Independent and joint effects of body mass index and metabolic health in mid- and late-life on all-cause mortality: a cohort study from the Swedish Twin Registry with a mean follow-up of 13 Years
Source: BMC Public Health. 2022 Apr 11;22:718. doi: 10.1186/s12889-022-13082-3 (PMC9004188; doi:10.1186/s12889-022-13082-3)
Supplement: Supplementary file 1 — Additional file 1: Additional files. Table S1. Effects of bodymass index and metabolic health status interaction on all-cause mortality whenCVD was included as a component for ascertaining metabolic health. Table S2. Effects of bodymass index and metabolic health status interaction on all-cause mortality, whenself-reports were excluded from the ascertainment of metabolic health status. Table S3. Effects of bodymass index and metabolic health status interaction on all-cause mortality, whenmetabolic health is defined as the absence of any metabolic abnormality. Table S4. Effects of body massindex and metabolic health status interaction on all-cause mortality, when waistcircumference was included in the ascertainment of metabolic health status, andmetabolic health is defined as ≤ two metabolic abnormalities. Table S5. Effects of body massindex and metabolic health status interaction on all-cause mortality when waistcircumference is included in the ascertainment of metabolic health status, andmetabolic health is defined as ≤ three metabolic abnormalities. Table S6. Effects of body massindex and metabolic health status interaction on all-cause mortality, when MHS isdefined by new criteria [1], the absence ofhypertension, high waist-hip ratios and diabetes. Table S7a. Effects of mid-life bodymass index and metabolic health status interaction on all-cause mortality, stratifiedby sex. Table S7b. Effects of late-life bodymass index and metabolic health status interaction on all-cause mortality, stratifiedby sex. [file 12889_2022_13082_MOESM1_ESM.docx]

**Additional files**

**Table S1**: Effects of body mass index and metabolic health status interaction on all-cause mortality when CVD was included as a component for ascertaining metabolic health.

| Age | BMI categories | MH status | Phenotype | n | Events | HR | 95% CI |
| --- | --- | --- | --- | --- | --- | --- | --- |
| Midlife (n=6252) | Normal weight | MH | MHN | 1878 | 183 | ref | |
|  |  | MU | MUN | 908 | 115 | **1.29** | **1.02 – 1.65** |
|  | Overweight | MH | MHOw | 1140 | 104 | 0.91 | 0.71 – 1.16 |
|  |  | MU | MUOw | 1441 | 202 | **1.29** | **1.06 – 1.58** |
|  | Obesity | MH | MHO | 210 | 21 | 1.02 | 0.67 -1.55 |
|  |  | MU | MUO | 675 | 108 | **1.75** | **1.38 – 2.23** |
| Late-life (n=6215) | Normal weight | MH | MHN | 1259 | 652 | ref | |
|  |  | MU | MUN | 1382 | 849 | **1.25** | **1.14 - 1.38** |
|  | Overweight | MH | MHOw | 832 | 373 | 0.87 | 0.78 - 0.99 |
|  |  | MU | MUOw | 1869 | 1066 | **1.24** | **1.13 - 1.37** |
|  | Obesity | MH | MHO | 163 | 87 | 1.09 | 0.88 - 1.35 |
|  |  | MU | MUOw | 674 | 392 | **1.46** | **1.30 - 1.65** |

Hazard ratios(HR) with 95% confidence intervals(CI) from Cox regression models of all-cause mortality in relation to interactive effects of body mass index (BMI) and metabolic health status (MHS) when CVD was added in the definition of MHS. Bold numbers indicate significance at the α=0.05 level.

Reference group was MHN. Models were adjusted for smoking, education, and sex.

Abbreviations: ref – reference group; MH – metabolically healthy; MU – metabolically unhealthy; MHN – metabolically healthy normal weight; MUN – metabolically unhealthy normal weight; MHOw – metabolically healthy overweight; MUOw – metabolically unhealthy overweight; MHO – metabolically healthy obesity; MUO – metabolically unhealthy obesity; CVD – cardiovascular disease; HDL high-density lipoprotein.

MH status is defined as ≤1 of the following: 1) hypertension 2) hyperglycaemia 3) triglyceridemia 4) low HDL 5) CVD

**Table S2**: Effects of body mass index and metabolic health status interaction on all-cause mortality, when self-reports were excluded from the ascertainment of metabolic health status.

| Age | BMI categories | MH status | Phenotype | n | Events | HR | 95% CI | HR | 95% CI |
| --- | --- | --- | --- | --- | --- | --- | --- | --- | --- |
|  |  |  |  |  |  | Model 7 | | Model 8 | |
| Midlife (n=6054) | Normal weight | MH | MHN | 2245 | 201 | ref | |  |  |
|  |  | MU | MUN | 443 | 54 | 1.26 | 0.92 - 1.74 | 1.20 | 0.87 - 1.65 |
|  | Overweight | MH | MHOw | 1573 | 144 | 0.97 | 0.78 - 1.20 | 0.94 | 0.76 – 1.17 |
|  |  | MU | MUOw | 935 | 125 | 1.25 | 1.00 - 1.57 | 1.16 | 0.93 – 1.46 |
|  | Obesity | MH | MHO | 364 | 30 | 0.95 | 0.65 - 1.39 | 0.87 | 0.60 – 1.28 |
|  |  | MU | MUO | 494 | 79 | **1.81** | **1.39 - 2.35** | **1.60** | **1.23 – 2.08** |
| Late-life (n=5120) | Normal weight | MH | MHN | 1593 | 672 | ref | |  |  |
|  |  | MU | MUN | 538 | 287 | **1.32** | **1.15 - 1.52** | **1.28** | **1.12 – 1.47** |
|  | Overweight | MH | MHOw | 1258 | 533 | 0.98 | 0.88 - 1.09 | 0.96 | 0.86 – 1.07 |
|  |  | MU | MUOw | 1038 | 516 | **1.26** | **1.12 - 1.40** | **1.19** | **1.06 – 1.33** |
|  | Obesity | MH | MHO | 267 | 124 | 1.15 | 0.96 - 1.39 | 1.08 | 0.89 - 1.30 |
|  |  | MU | MUOw | 426 | 213 | **1.47** | **1.26 - 1.72** | **1.39** | **1.19 – 1.62** |

Hazard ratios(HR) with 95% confidence intervals(CI) from Cox regression models of all-cause mortality in relation to interactive effects of body mass index (BMI) and metabolic health status (MHS) when self-report use of diabetic medications, lipid-lowering medications and diagnosis of diabetes were excluded from the ascertainment of MHS (sample size n=11,174). Bold numbers indicate significance at the α=0.05 level.

Reference group was MHN. Model 7 was adjusted for smoking, education, sex. Model 8 was further adjusted for the CVD.

Abbreviations: ref – reference group; MH – metabolically healthy; MU – metabolically unhealthy; MHN – metabolically healthy normal weight; MUN – metabolically unhealthy normal weight; MHOw – metabolically healthy overweight; MUOw – metabolically unhealthy overweight; MHO – metabolically healthy obesity; MUO – metabolically unhealthy obesity; CVD – cardiovascular disease; HDL high-density lipoprotein.

MU status is defined as ≤2 of the following: 1) hypertension 2) hyperglycaemia 3) triglyceridemia 4) low HDL.

**Table S3**: Effects of body mass index and metabolic health status interaction on all-cause mortality, when metabolic health is defined as the absence of any metabolic abnormality.

| Age | BMI categories | MH status | Phenotype | n | Events | HR | 95% CI | HR | 95% CI |
| --- | --- | --- | --- | --- | --- | --- | --- | --- | --- |
|  |  |  |  |  |  | Model 7 | | Model 8 | |
| Midlife (n=6252) | Normal weight | MH | MHN | 926 | 70 | ref |  | ref | |
|  |  | MU | MUN | 1860 | 228 | 1.14 | 0.86 – 1.50 | 1.06 | 0.80 - 1.39 |
|  | Overweight | MH | MHOw | 400 | 30 | 0.94 | 0.61 – 1.46 | 0.94 | 0.61 - 1.45 |
|  |  | MU | MUOw | 2181 | 276 | 1.16 | 0.88 – 1.52 | 1.04 | 0.79 - 1.38 |
|  | Obesity | MH | MHO | *59* | *10* | **2.41** | **1.29 – 4.50** | **2.29** | **1.23 - 4.35** |
|  |  | MU | MUO | 826 | 119 | **1.53** | **1.13 – 2.06** | 1.31 | 0.97 - 1.78 |
| Late-life (n=6215) | Normal weight | MH | MHN | 359 | 142 | ref | | ref | |
|  |  | MU | MUN | 2318 | 1359 | **1.25** | **1.06 – 1.48** | 1.13 | 0.96 - 1.33 |
|  | Overweight | MH | MHOw | 178 | 68 | 1.06 | 0.80 – 1.40 | 1.01 | 0.77 - 1.33 |
|  |  | MU | MUOw | 2523 | 1371 | **1.22** | **1.04 – 1.44** | 1.08 | 0.91 - 1.27 |
|  | Obesity | MH | MHO | *27* | *15* | **2.04** | **1.27 – 3.28** | **1.83** | **1.13 - 2.99** |
|  |  | MU | MUO | 810 | 464 | **1.48** | **1.24 – 1.77** | **1.28** | **1.07 - 1.53** |

Hazard ratios(HR) with 95% confidence intervals(CI) from Cox regression models of all-cause mortality in relation to interactive effects of body mass index (BMI) and metabolic health status (MHS), when metabolic health is defined as the absence of metabolic abnormality. Bold numbers indicate significance at the α =0.05 level. Numbers in italics bring attention to low cell number.

Reference group was MHN. Model 7 was adjusted for smoking, education, sex. Model 8 was further adjusted for the CVD

Abbreviations: ref – reference group; MH – metabolically healthy; MU – metabolically unhealthy; MHN – metabolically healthy normal weight; MUN – metabolically unhealthy normal weight; MHOw – metabolically healthy overweight; MUOw – metabolically unhealthy overweight; MHO – metabolically healthy obesity; MUO – metabolically unhealthy obesity; CVD – cardiovascular disease; HDL high-density lipoprotein.

Metabolic healthy status is defined as the absence of the following: 1) hypertension 2) hyperglycaemia 3) triglycerideamia 4) low HDL.

**Table S4:** Effects of body mass index and metabolic health status interaction on all-cause mortality, when waist circumference was included in the ascertainment of metabolic health status, and metabolic health is defined as ≤ two metabolic abnormalities.

| Age | BMI categories | MH status | Phenotype | n | Events | HR | 95% CI | HR | 95% CI |
| --- | --- | --- | --- | --- | --- | --- | --- | --- | --- |
|  |  |  |  |  |  | Model 7 | | Model 8 | |
| Midlife (n=6236) | Normal weight | MH | MHN | 1684 | 166 | ref |  | ref | |
|  |  | MU | MUN | 1099 | 132 | 1.22 | 0.96 – 1.53 | 1.15 | 0.91 - 1.45 |
|  | Overweight | MH | MHOw | 537 | 38 | 0.75 | 0.52 – 1.08 | 0.76 | 0.53 - 1.10 |
|  |  | MU | MUOw | 2034 | 267 | 1.19 | 0.98 – 1.45 | 1.12 | 0.92 - 1.36 |
|  | Obesity | MH | MHO | *59* | *10* | **2.37** | **1.30 – 4.32** | **2.34** | **1.27 - 4.30** |
|  |  | MU | MUO | 823 | 117 | **1.48** | **1.17 – 1.87** | **1.33** | **1.04 - 1.67** |
| Late-life (n=6201) | Normal weight | MH | MHN | 1257 | 654 | ref |  | Ref | |
|  |  | MU | MUN | 1413 | 840 | **1.18** | **1.08 – 1.30** | 1.10 | 1.00 - 1.21 |
|  | Overweight | MH | MHOw | 313 | 136 | 0.88 | 0.74 – 1.04 | 0.86 | 0.72 - 1.03 |
|  |  | MU | MUOw | 2381 | 1298 | **1.12** | **1.02 – 1.23** | 1.04 | 0.95 - 1.13 |
|  | Obesity | MH | MHO | *29* | *16* | **1.80** | **1.17 – 2.78** | **1.70** | **1.09 - 2.64** |
|  |  | MU | MUOw | 808 | 463 | **1.33** | **1.19 – 1.49** | **1.21** | **1.08 - 1.36** |

Hazard ratios(HR) with 95% confidence intervals(CI) from Cox regression models of all-cause mortality in relation to interactive effects of body mass index (BMI) and metabolic health status (MHS), when waist circumference was included in the MHS definition. Bold numbers indicate significance at the α=0.05 level. Numbers in italics bring attention to low cell number.

Reference group was MHN. Model 7 was adjusted for smoking, education, sex. Model 8 was further adjusted for the CVD.

Abbreviations: ref – reference group; MH – metabolically healthy; MU – metabolically unhealthy; MHN – metabolically healthy normal weight; MUN – metabolically unhealthy normal weight; MHOw – metabolically healthy overweight; MUOw – metabolically unhealthy overweight; MHO – metabolically healthy obesity; MUO – metabolically unhealthy obesity; CVD – cardiovascular disease; HDL high-density lipoprotein; CVD – cardiovascular disease

MU status is defined as ≤2 of the following: 1) hypertension 2) hyperglycaemia 3) triglyceridemia 4) low HDL 4) high waist circumference

**Table S5:** Effects of body mass index and metabolic health status interaction on all-cause mortality when waist circumference is included in the ascertainment of metabolic health status, and metabolic health is defined as ≤ three metabolic abnormalities.

| Age | BMI categories | MH status | Phenotype | n | Events | HR | 95% CI | HR | 95% CI |
| --- | --- | --- | --- | --- | --- | --- | --- | --- | --- |
|  |  |  |  |  |  | Model 7 | | Model 8 | |
| Midlife (n=6236) | Normal weight | MH | MHN | 2381 | 253 | ref |  | ref | |
|  |  | MU | MUN | 402 | 45 | 1.14 | 0.81 – 1.61 | 1.05 | 0.75 - 1.49 |
|  | Overweight | MH | MHOw | 1510 | 154 | 0.90 | 0.74 – 1.10 | 0.89 | 0.73 - 1.10 |
|  |  | MU | MUOw | 1061 | 151 | **1.27** | **1.03 – 1.56** | 1.16 | 0.94 - 1.43 |
|  | Obesity | MH | MHO | 316 | 34 | 1.08 | 0.77 – 1.52 | 1.04 | 0.74 - 1.46 |
|  |  | MU | MUO | 566 | 93 | **1.64** | **1.29 – 2.08** | **1.45** | **1.14 - 1.86** |
| Late-life (n=6201) | Normal weight | MH | MHN | 2075 | 1141 | ref |  | ref | |
|  |  | MU | MUN | 595 | 353 | **1.30** | **1.15 – 1.46** | **1.21** | **1.07 - 1.36** |
|  | Overweight | MH | MHOw | 1341 | 693 | 0.94 | 0.86 -1.02 | 0.92 | 0.84 - 1.01 |
|  |  | MU | MUOw | 1353 | 741 | **1.20** | **1.10 – 1.32** | **1.11** | **1.02 - 1.22** |
|  | Obesity | MH | MHO | 303 | 179 | 1.13 | 0.97 – 1.31 | 1.07 | 0.93 - 1.24 |
|  |  | MU | MUOw | 534 | 300 | **1.43** | **1.26 – 1.62** | **1.32** | **1.16 - 1.50** |

Hazard ratios(HR) with 95% confidence intervals(CI) from Cox regression models of all-cause mortality in relation to interactive effects of body mass index (BMI) and metabolic health status (MHS), when waist circumference was included in the MHS definition. Bold numbers indicate significance at the α=0.05 level.

Reference group was MHN. Model 7 was adjusted for smoking, education, sex. Model 8 was further adjusted for the CVD.

Abbreviations: ref – reference group; MH – metabolically healthy; MU – metabolically unhealthy; MHN – metabolically healthy normal weight; MUN – metabolically unhealthy normal weight; MHOw – metabolically healthy overweight; MUOw – metabolically unhealthy overweight; MHO – metabolically healthy obesity; MUO – metabolically unhealthy obesity; CVD – cardiovascular disease; HDL high-density lipoprotein; CVD – cardiovascular disease

MU status is defined as ≤3 of the following: 1) hypertension 2) hyperglycaemia 3) triglyceridemia 4) low HDL 4) high waist circumference.

**Table S6:** Effects of body mass index and metabolic health status interaction on all-cause mortality, when MHS is defined by new criteria [1], the absence of hypertension, high waist-hip ratios and diabetes.

| Age | BMI categories | MH status | Phenotype | n | Events | HR | 95% CI | HR | 95% CI |
| --- | --- | --- | --- | --- | --- | --- | --- | --- | --- |
|  |  |  |  |  |  | Model 7 | | Model 8 | |
| Midlife (n=6236) | Normal weight | MH | MHN | 1339 | 114 | ref |  | ref | |
|  |  | MU | MUN | 1412 | 182 | 1.03 | 0.82 – 1.30 | 0.97 | 0.77 – 1.23 |
|  | Overweight | MH | MHOw | 812 | 62 | 0.78 | 0.57 – 1.07 | 0.76 | 0.56 – 1.05 |
|  |  | MU | MUOw | 1738 | 240 | 1.14 | 0.91 – 1.42 | 1.05 | 0.83 – 1.32 |
|  | Obesity | MH | MHO | ***150*** | ***14*** | 1.05 | 0.60 – 1.83 | 0.97 | 0.56 – 1.69 |
|  |  | MU | MUO | 724 | 111 | **1.48** | **1.14 – 1.92** | 1.30 | 1.00 – 1.70 |
| Late-life (n=6201) | Normal weight | MH | MHN | 561 | 233 | ref |  |  | |
|  |  | MU | MUN | 2097 | 1252 | 1.12 | 0.98 – 1.29 | 1.05 | 0.92 – 1.21 |
|  | Overweight | MH | MHOw | 381 | 156 | 1.12 | 0.92 – 1.35 | 1.07 | 0.89 – 1.30 |
|  |  | MU | MUOw | 2298 | 1272 | 1.10 | 0.96 – 1.26 | 1.01 | 0.88 – 1.16 |
|  | Obesity | MH | MHO | ***65*** | ***34*** | **1.19** | **1-36 – 2.52** | **1.67** | **1.23 – 2.28** |
|  |  | MU | MUOw | 771 | 445 | **1.32** | **1.14 – 1.55** | **1.19** | **1.01 – 1.39** |

Hazard ratios(HR) with 95% confidence intervals(CI) from Cox regression models of all-cause mortality in relation to interactive effects of body mass index (BMI) and metabolic health status (MHS) with new criteria. Bold numbers indicate significance at the α=0.05 level. Italicized numbers bring attention to low cell number.

Reference group was MHN. Model 7 was adjusted for smoking, education, sex. Model 8 was further adjusted for the CVD.

Abbreviations: ref – reference group; MH – metabolically healthy; MU – metabolically unhealthy; MHN – metabolically healthy normal weight; MUN – metabolically unhealthy normal weight; MHOw – metabolically healthy overweight; MUOw – metabolically unhealthy overweight; MHO – metabolically healthy obesity; MUO – metabolically unhealthy obesity; CVD – cardiovascular disease; HDL high-density lipoprotein; CVD – cardiovascular disease

Metabolic health is defined as the absence of 1) systolic BP more than 130 mmHg, 2) waist-hip ratios more than 0.95 for women and more than 1.03 in men, 3) no prevalent diabetes.

**Table S7a:** Effects of mid-life body mass index and metabolic health status interaction on all-cause mortality, stratified by sex.

| Sex | BMI categories | MH status | Phenotype | n | Events | HR | 95% CI | HR | 95% CI |
| --- | --- | --- | --- | --- | --- | --- | --- | --- | --- |
|  |  |  |  |  |  | Model 7 | | Model 8 | |
| Females (n= 3498) | Normal weight | MH | MHN | 1465 | 135 | ref |  | ref | |
|  |  | MU | MUN | 350 | 37 | 1.14 | 0.76 – 1.70 | 1.07 | 0.71 – 1.61 |
|  | Overweight | MH | MHOw | 786 | 67 | 0.87 | 0.65 – 1.16 | 0.86 | 0.64 – 1.15 |
|  |  | MU | MUOw | 407 | 44 | 1.23 | 0.88 – 1.74 | 1.16 | 0.83 – 1.63 |
|  | Obesity | MH | MHO | 204 | 16 | 1.03 | 0.63 – 1.67 | 0.99 | 0.61 – 1.60 |
|  |  | MU | MUO | 286 | 36 | **1.46** | **1.01 – 2.11** | 1.32 | 0.91 – 1.91 |
| Males (n= 2754) | Normal weight | MH | MHN | 713 | 90 | ref |  | ref | |
|  |  | MU | MUN | 258 | 36 | 1.41 | 0.95 -2.09 | 1.30 | 0.87 – 1.94 |
|  | Overweight | MH | MHOw | 668 | 78 | 1.00 | 0.73 – 1.36 | 0.96 | 0.70 – 1.31 |
|  |  | MU | MUOw | 720 | 117 | **1.44** | **1.09 – 1.89** | **1.33** | **1.00 – 1.77** |
|  | Obesity | MH | MHO | 112 | 18 | 1.26 | 0.77 – 2.05 | 1.20 | 0.73 – 1.97 |
|  |  | MU | MUOw | 283 | 59 | **2.09** | **1.50 – 2.91** | **1.86** | **1.32 – 2.63** |

Hazard ratios with 95% confidence intervals of all-cause mortality in relation to the interaction between body mass index categories (BMI) and metabolic health status (MHS) stratified by sex for the midlife group. Bold numbers indicate significance at the α=0.05 level.

Reference group was MHN – metabolically healthy normal weight. Model 7 was adjusted for smoking, education, sex. Model 8 was further adjusted for the CVD.

Abbreviations: CVD - history of cardiovascular disease, MUN – metabolically unhealthy normal, MHOw – metabolically healthy overweight, MUOw – metabolically unhealthy overweight, MHO – metabolically healthy obesity and MUO – metabolically unhealthy obesity.

Normal weight is defined as having BMI 18.5 – 24.9 kg/m^2^; overweight 25 – 29kg/m^2^; obesity ≥30 kg/m^2^. MU is defined as having ≥ two abnormal metabolic abnormalities: 1) hypertension 2) hyperglycaemia 3) triglyceridemia 4) low HDL

**Table S7b:** Effects of late-life body mass index and metabolic health status interaction on all-cause mortality, stratified by sex.

| Sex | BMI categories | MH status | Phenotype | n | Events | HR | 95% CI | HR | 95% CI |
| --- | --- | --- | --- | --- | --- | --- | --- | --- | --- |
|  |  |  |  |  |  | Model 7 | | Model 8 | |
| Females (n= 3318) | Normal weight | MH | MHN | 1017 | 527 | ref |  | ref | |
|  |  | MU | MUN | 463 | 259 | **1.19** | **1.03 – 1.37** | 1.14 | 0.98 – 1.32 |
|  | Overweight | MH | MHOw | 688 | 346 | 0.93 | 0.82 – 1.05 | 0.91 | 0.80 – 1.04 |
|  |  | MU | MUOw | 635 | 303 | 1.13 | 0.99 – 1.30 | 1.08 | 0.94 – 1.23 |
|  | Obesity | MH | MHO | 199 | 113 | 1.07 | 0.89 – 1.29 | 1.02 | 0.85 – 1.23 |
|  |  | MU | MUO | 316 | 163 | **1.39** | **1.16 – 1.66** | **1.30** | **1.09 – 1.56** |
| Males (n= 2897) | Normal weight | MH | MHN | 783 | 464 | ref |  | ref | |
|  |  | MU | MUN | 414 | 251 | **1.21** | **1.05 – 1.40** | 1.10 | 0.95 – 1.28 |
|  | Overweight | MH | MHOw | 594 | 316 | 0.94 | 0.82 – 1.08 | 0.91 | 0.79 – 1.04 |
|  |  | MU | MUOw | 784 | 474 | **1.25** | **1.10 – 1.41** | **1.12** | 0.99 – 1.27 |
|  | Obesity | MH | MHO | 103 | 66 | 1.21 | 0.95 – 1.55 | 1.15 | 0.90 – 1.47 |
|  |  | MU | MUOw | 219 | 137 | **1.50** | **1.24 – 1.80** | **1.33** | **1.11 – 1.61** |

Hazard ratios with 95% confidence intervals of all-cause mortality in relation to the interaction between body mass index categories (BMI) and metabolic health status (MHS) stratified by sex for the late-life group. Reference group is MHN – metabolically healthy normal weight.

Model 7 was adjusted for smoking, education, sex. Model 8 was further adjusted for the CVD.

Abbreviations: CVD - history of cardiovascular disease, MUN – metabolically unhealthy normal, MHOw – metabolically healthy overweight, MUOw – metabolically unhealthy overweight, MHO – metabolically healthy obesity and MUO – metabolically unhealthy obesity.

Normal weight is defined as having BMI 18.5 – 24.9 kg/m^2^; overweight 25 – 29kg/m^2^; obesity ≥30 kg/m^2^. MU is defined as having ≥ two abnormal metabolic abnormalities: 1) hypertension 2) hyperglycaemia 3) triglyceridemia 4) low HDL

Reference

1. Zembic A, Eckel N, Stefan N, Baudry J, Schulze MB: **An Empirically Derived Definition of Metabolically Healthy Obesity Based on Risk of Cardiovascular and Total Mortality**. *JAMA Netw Open* 2021, **4**(5):e218505.
